# Perceived Impact of Early On-Call Exposure on General Medical Council Outcomes Among Graduates: A Project in an English Foundation School

**DOI:** 10.7759/cureus.117865

**Published:** 2026-10-13

**Authors:** Hayder Noori, Yosef Mustafa, Samuel Audet, Eshtar Hamid

**Affiliations:** 1 Medical Education, School of Medicine, Faculty of Health, Medicine and Social Care, Anglia Ruskin University, Chelmsford, GBR; 2 Medical Education, Southend University Hospital NHS Foundation Trust, Southend, GBR; 3 Dermatology, University College London Hospital, London, GBR

**Keywords:** foundation year 1 doctors, medical students, mixed-methods research, night shift, on-call experience, on-call shadowing, out-of-hours, preparedness for practice, transition to clinical practice, undergraduate medical education

## Abstract

Background: Transitioning from medical school to Foundation training is widely recognised as a challenging stage, with new doctors often reporting underpreparedness for clinical responsibilities. Early exposure to on-call shifts during undergraduate training may help address this gap.

Aim: This study evaluated Foundation doctors' perceptions of how early on-call experience during United Kingdom medical school related to their preparedness and to their development of professional knowledge, skills, and values and behaviours, as defined in the General Medical Council Outcomes for Graduates.

Methods: A convergent parallel mixed-methods design was used. A survey of 71 Foundation Year 1 doctors in an English Foundation School achieved 19 responses (26.7%). Quantitative data were collected using Likert-scale questions and analysed descriptively and with exploratory one-sample Wilcoxon signed-rank tests against a neutral response, while qualitative data from open-ended questions underwent thematic analysis.

Results: Of the 19 participants, 18 (94.7%) had undertaken on-call placements, predominantly in years 4 and 5. Perceptions were generally positive, with the highest mean Likert scores for knowledge (3.56) and skills (3.44), and lower scores for preparedness (3.22) and professional values (3.22); however, none of the four statements differed significantly from neutral (p = 0.057-0.235). Thematic analysis highlighted perceived benefits, including exposure to acute cases, improved confidence, and enhanced decision-making. Challenges included variability in supervision, limited teaching during night shifts, and difficulty adapting to out-of-hours schedules.

Conclusion: Respondents generally perceived undergraduate on-call exposure positively, particularly for knowledge and skills, although these findings were not statistically significant and views on professional values and behaviours were less consistent. Perceived educational value varied according to supervision and context. Larger comparative studies are needed to determine whether structured on-call placements improve preparedness.

## Introduction

The transition from being a medical student to becoming a Foundation trainee is widely recognised as one of the most stressful phases in a doctor’s professional journey. This period is characterised by a significant increase in clinical responsibilities, the expectation to make more autonomous decisions, and the need to apply theoretical knowledge in real-life, often high-pressure situations. Alongside these demands, new doctors are required to demonstrate a growing level of confidence, competence, and professionalism as they take on the care of patients in a more independent capacity [1]. The so-called “July/August effect”, where patient outcomes are shown to dip during the annual changeover of junior doctors, is often cited as evidence that this transition remains problematic and warrants ongoing attention [2].

The General Medical Council (GMC) has established guidance to ensure doctors are adequately prepared to provide safe, effective, and high-quality care within the UK healthcare system. Central to this is the Generic Professional Capabilities Framework, which defines the essential competencies all doctors must develop and maintain throughout their careers [3]. In line with this framework, the GMC has outlined specific Outcomes for Graduates, ensuring that newly qualified doctors possess foundational abilities across three domains: professional values and behaviours, professional skills, and professional knowledge [4]. These outcomes provide a baseline for ongoing professional growth during postgraduate training and beyond, emphasising the importance of demonstrating competence in each domain at the point of qualification.

Research has shown that Foundation trainees often demonstrate a lack of preparedness and confidence when faced with the realities of making medical decisions in clinical settings [5]. This lack of preparedness has been linked to longstanding criticisms that United Kingdom (UK) medical curricula were overly theoretical, with insufficient focus on clinical preparedness. In response to these concerns, the publication of Tomorrow’s Doctors in 1993 [6] and subsequent reforms pushed medical schools towards integrated, patient-centred training, marking the beginning of a sustained shift towards better preparing graduates for real-world clinical practice.

Despite substantial reforms in UK medical education, particularly the increased focus on early clinical exposure and practical skills training, many graduates continue to feel underprepared for the demands of the Foundation Programme. This underpreparedness extends beyond clinical decision-making to key professional behaviours, including effective communication, teamwork, and the demonstration of professional values and attitudes [7,8]. Such deficiencies can have important implications for patient safety, as well as for the confidence, well-being, and professional identity development of newly qualified doctors.

In a national survey assessing the preparedness of new Foundation doctors, both trainees and their supervisors concluded that increased time spent shadowing experienced doctors prior to starting clinical duties would significantly enhance preparedness for practice [5]. While shadowing offers valuable observational learning, one crucial aspect of real-world clinical exposure that remains underrepresented in many undergraduate programmes is active participation in on-call shifts, defined as extended duty periods that may take the form of either long day shifts or night shifts. These experiences expose students to the unpredictable, high-pressure, and high-responsibility environments that they will inevitably encounter as junior doctors [9].

Early and structured integration of on-call experiences into medical school curricula could therefore play a vital role in helping students develop not only the clinical knowledge and skills outlined in the GMC Outcomes for Graduates, but also the professional behaviours and decision-making capabilities essential for safe and confident practice [10].

This study aimed to answer the question: what is the perceived impact of early exposure to on-call shifts during a UK undergraduate medicine programme on Foundation doctors' preparedness and on their development of professional knowledge, skills, and values and behaviours, the three domains of the GMC Outcomes for Graduates? It was designed to evaluate Foundation doctors' self-reported perceptions of preparedness for the Foundation Programme in relation to these domains. This study assessed self-reported perceptions and did not directly measure attainment of the GMC Outcomes for Graduates. In addition, it aimed to describe how on-call shifts are currently integrated into undergraduate medical curricula, including whether they are a formal, structured component of training, the stage at which they are introduced, and whether they are mandatory or optional. The study employed a convergent parallel mixed-methods design to collect both quantitative (closed-ended) and qualitative (open-ended) data via a structured survey.

## Materials and methods

The survey was distributed to all 71 UK medical graduates working as Foundation Year 1 (FY1) doctors in an English Foundation School during July to August 2025, at the transition to Foundation Year 2 (FY2), using a total population sampling approach in which every eligible doctor at the site was invited to participate. Of these, 19 doctors responded, giving a response rate of 26.7%. Eligible participants were UK-trained FY1s, while non-UK graduates and FY2s were excluded. This group was chosen to capture insights from doctors completing their first year of practice, ensuring relevance to UK curricula.

Because this study was descriptive and exploratory rather than designed to test a pre-specified hypothesis, no formal sample size calculation was performed. Adequacy of the achieved sample was instead judged on two grounds: for the quantitative component, the aim was to describe patterns within this single-site cohort rather than to estimate population parameters; for the qualitative component, sample adequacy was judged by information power rather than a fixed target size, and the consistent, well-illustrated themes identified across the 19 respondents' open-ended answers supported reasonable information power despite the modest sample [11].

Data were collected using a 12-question Microsoft Forms survey (Appendices), developed by the lead author specifically for this study to address the research questions rather than adapted from an existing published questionnaire. Items were mapped to the three GMC Outcomes for Graduates domains [4]. The survey combined Likert-scale items to assess preparedness, skills, knowledge, and professional behaviours with open-ended questions to explore individual experiences. Participants provided consent by ticking a checkbox at the start of the survey before proceeding to the questions. The survey also investigated how on-call shifts were integrated into participants' undergraduate curricula, including their timing and whether they were mandatory or optional, and allowed participants without on-call experience to comment on how such exposure might have influenced their preparedness. The survey was reviewed and approved by the Anglia Ruskin University School Research Ethics Panel as part of the ethical approval process.

Incentives in research have been shown to improve response rates [12]. Therefore, to encourage engagement and acknowledge participants' time constraints, all respondents were entered into a random lottery with the chance to win one of three Amazon gift vouchers: £60 for first prize, £40 for second, and £20 for third.

Quantitative data were analysed descriptively using graphs and narrative summaries. As an exploratory analysis, a one-sample Wilcoxon signed-rank test was applied to each Likert statement to assess whether responses differed from a neutral response. A non-parametric test was chosen because 18 responses per statement were insufficient to assume normality. Analyses were performed using Microsoft Excel (Microsoft Corporation, Redmond, Washington). Responses to the two open-ended questions were analysed thematically by the author, who read all responses, developed codes iteratively, and grouped recurring patterns into themes. Coding was performed by a single researcher without independent second coding. Quantitative and qualitative data were analysed separately and integrated at the interpretation stage, following a convergent parallel design. For each of the four constructs (preparedness, professional values and behaviours, professional skills, and professional knowledge), the Likert-scale results (mean score and Wilcoxon test outcome) were compared with the qualitative themes relating to that construct to identify areas of convergence, expansion, or divergence between the two data types. This comparison is presented in the Discussion [13].

Ethical approval was obtained from the Anglia Ruskin University School Research Ethics Panel, and the study was conducted in coordination with the Foundation School. Confidentiality was maintained by anonymising survey responses after the lottery was completed. Raw data cannot be shared because participants consented only to their responses being used in the report and future publications, not to public data sharing.

## Results

Responses were gathered from 19 of 71 Foundation doctors, representing a 26.7% response rate; 18 (94.7%) had early exposure to on-call shifts. They had predominantly attended placements in years 4 and 5, with some attending in years 2 and 3 (Figure 1). This reflects a broad range of pre-FY1 exposure. Of the 18 participants with on-call experience, 15 (83.3%) had mandatory on-call placements, while these were optional for the remaining three (16.7%) (Figure 2). 

**Figure 1:**
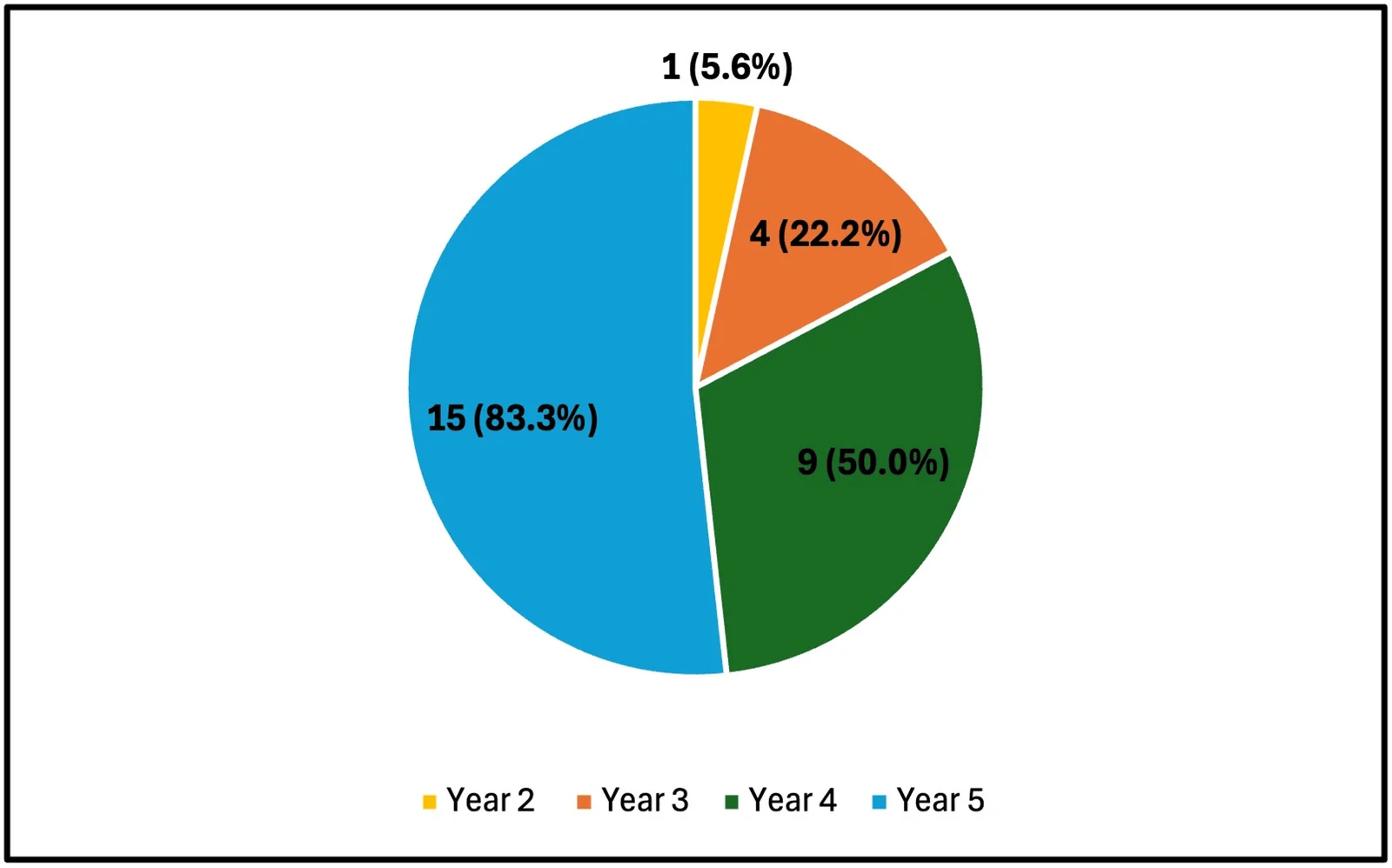
Timing of on-call placement exposure among students who attended placements (n = 18). Data presented as n (%) of participants attending each academic year. No inferential statistical tests were applied. Data are descriptive only.

**Figure 2:**
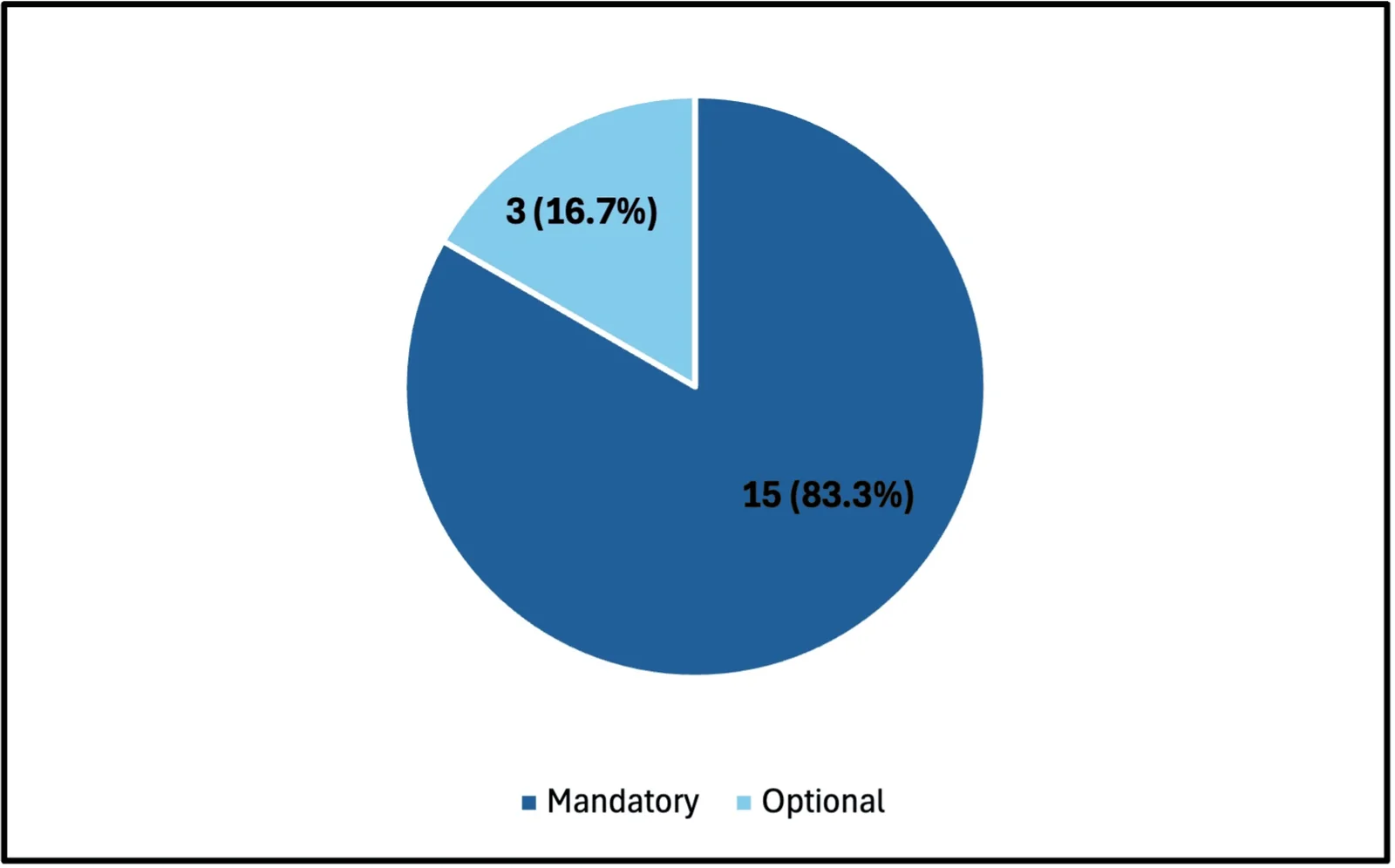
Proportion of students for whom on-call placement was mandatory versus optional (n = 18). Data are presented as n (%).

Quantitative data from 18 participants who attended on-call placements were gathered using four Likert-scale questions (1 = strongly disagree to 5 = strongly agree). As shown in Figure 3, responses to the statement “Early on-call shift exposure increased my preparedness for FY1” were generally positive, although 7 of the 18 (38.9%) participants selected a neutral response, while 2 (11%) disagreed. A similar pattern was observed for the statement “Early exposure to on-call shifts positively shaped my professional values and behaviour”, where options 3 and 4 (neutral and agree) received five responses each (27.8%).

The statements “Early exposure to on-call shifts contributed to the development of my professional skills” and “Early exposure to on-call shifts enhanced my professional knowledge” showed more consistently positive responses. Notably, 11 of the 18 (61.1%) participants selected options 4 or 5 (agree or strongly agree) for the question on professional knowledge, compared with 7-9 participants (38.9-50.0%) selecting these options for the other three questions (Figure 3).

**Figure 3:**
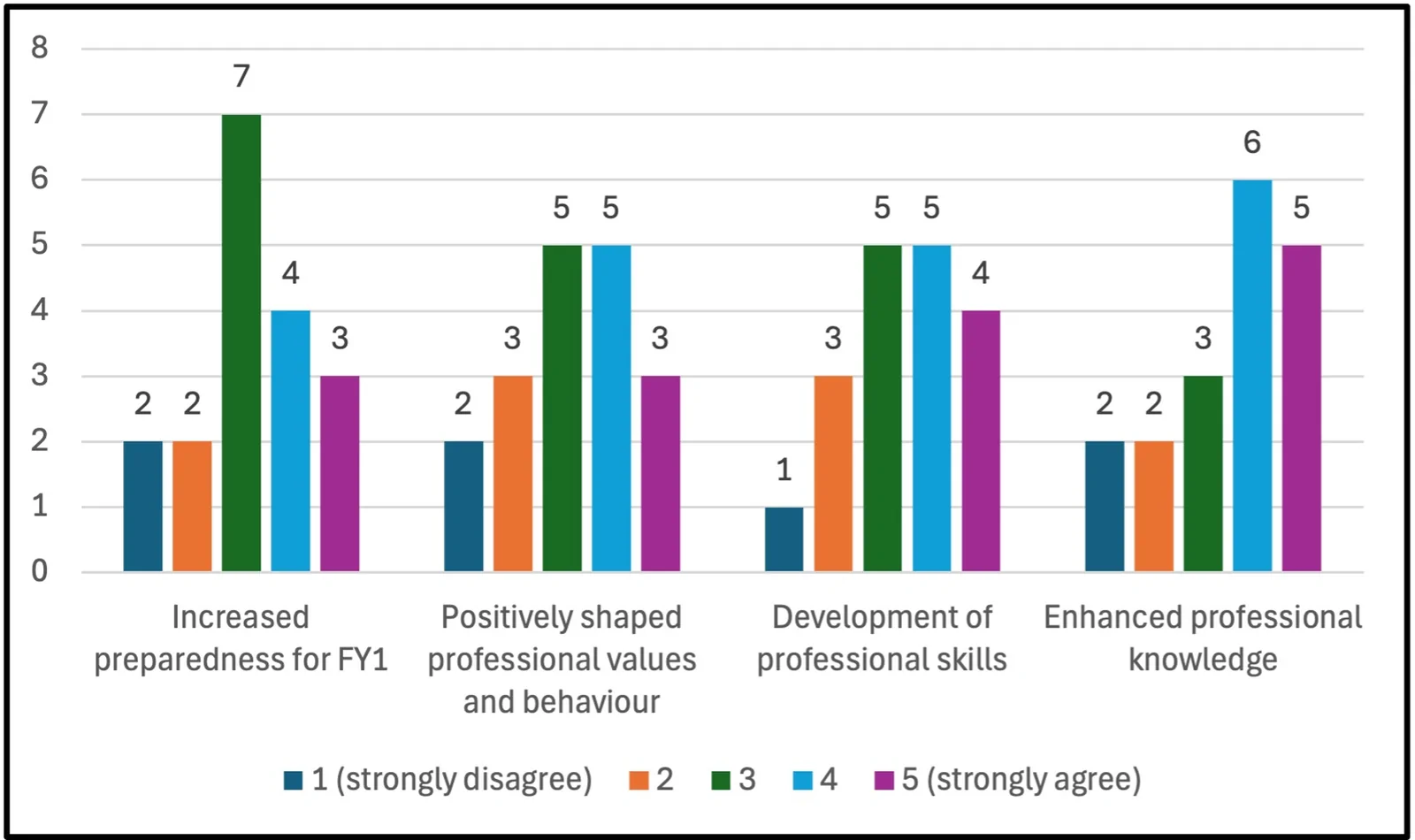
Distribution of Likert-scale responses (1 = strongly disagree to 5 = strongly agree) to four statements on the perceived impact of early on-call exposure (n = 18 per statement). Data are presented as n (frequency) of respondents selecting each option.

The mean scores for the four Likert-scale statements showed an overall favourable pattern of responses (Figure 4). The statements regarding preparedness for FY1 and perceived impact on professional values and behaviour each had an average score of 3.22, while professional skills scored higher at 3.44 and professional knowledge the highest at 3.56. Overall, these results suggest a broadly positive perception of early on-call shift exposure, with the strongest perceived impact reported in the development of professional skills and knowledge.

**Figure 4:**
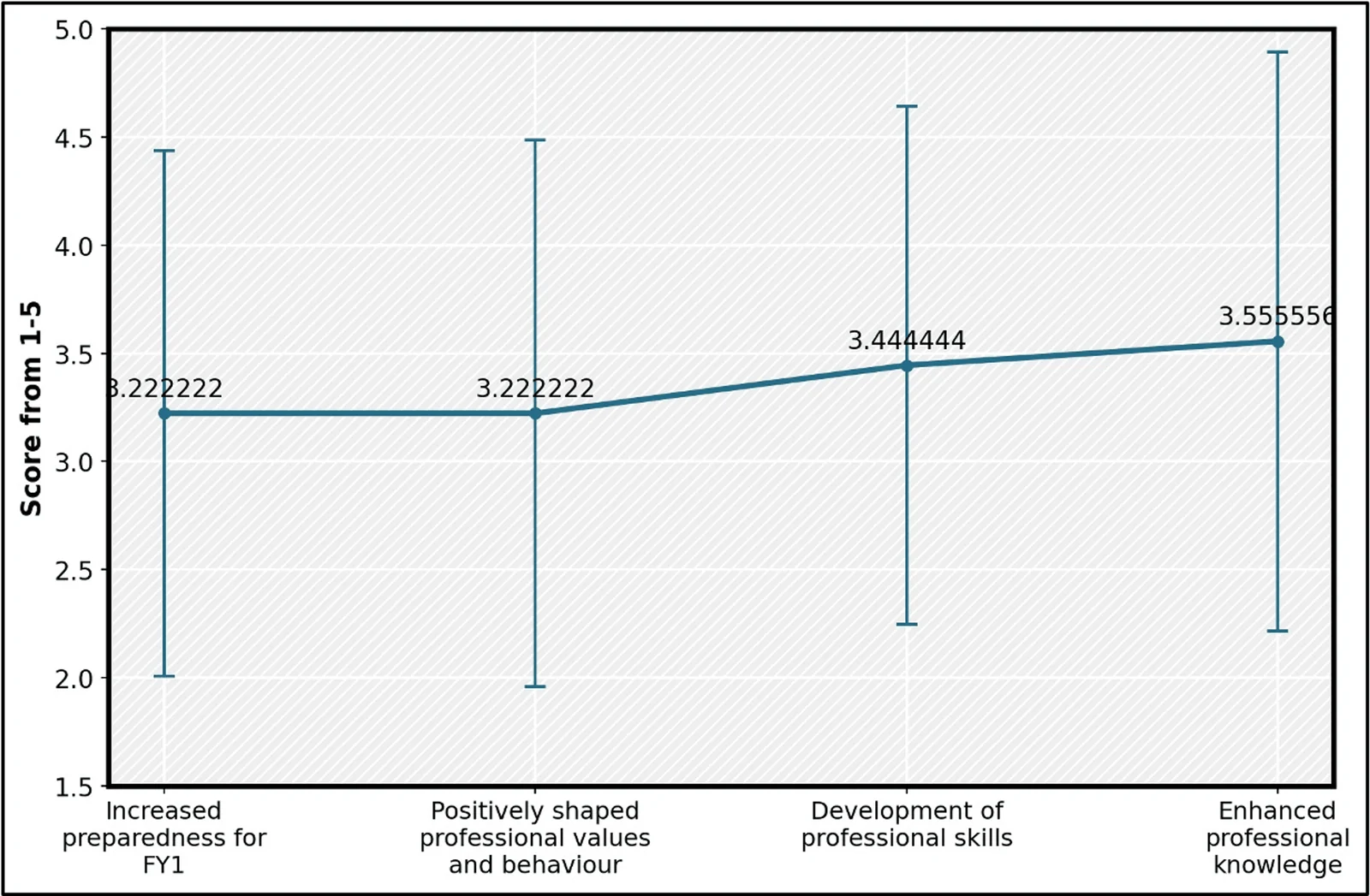
Mean Likert-scale scores for each statement on early on-call exposure (n = 18 per statement). Data are presented as mean ± standard deviation: preparedness for FY1: 3.22 ± 1.22; professional values and behaviour: 3.22 ± 1.26; professional skills: 3.44 ± 1.20; professional knowledge: 3.56 ± 1.34.

To determine whether these positive trends reflected statistically significant scores compared with a neutral reference score, a one-sample Wilcoxon signed-rank test was applied to each statement (Table 1). This non-parametric test was selected as 18 respondents per statement were insufficient to assume normally distributed data; therefore, the median of the ranked responses was compared against a fixed value. A reference value of 3 was used, as it corresponds to the neutral midpoint of 1-5 on the Likert scale, with an alternative hypothesis of the median being greater than neutral. The test revealed that none of the four statements reached statistical significance (p < 0.05) against the neutral reference. The statements on development of professional skills (p = 0.065) and enhanced professional knowledge (p = 0.057) had the lowest p-values, while those on increased preparedness for FY1 (p = 0.232) and positively shaped professional values/behaviour (p = 0.235) had higher values. Therefore, the inferential findings do not support the suggested benefits of early on-call exposure, although the results do support a broadly positive perception at a descriptive level. The small sample size (n=18 per statement) limits statistical power, so the study may have been unable to detect modest deviations from the neutral response.

**Table 1:** One-sample Wilcoxon signed-rank test results for each Likert-scale statement, tested against a neutral reference value of 3 (n = 18 per statement).

Statement	n	Mean	Median	SD	Wilcoxon vs 3 (p-value)
Increased preparedness for FY1	18	3.22	3.0	1.22	0.232
Positively shaped professional values/behaviour	18	3.22	3.0	1.26	0.235
Development of professional skills	18	3.44	3.5	1.20	0.065
Enhanced professional knowledge	18	3.56	4.0	1.34	0.057

The total frequency of quantitative responses indicates a generally positive perception of early on-call shift exposure. When considering the four Likert-scale questions collectively, the distribution of responses shows that only a small proportion of participants strongly disagreed (7/72, 9.7%) or disagreed (10/72, 13.9%) with the statements. In contrast, neutral or favourable views were more common, with 20 (27.8%) selecting option 3, another 20 (27.8%) selecting option 4, and 15 (20.8%) strongly agreeing. Taken together, these results suggest that most responses perceived early on-call experiences as beneficial, although the strength of agreement varied (Figure 5). No inferential statistical test was applied to the pooled responses because these included repeated responses from the same participants.

**Figure 5:**
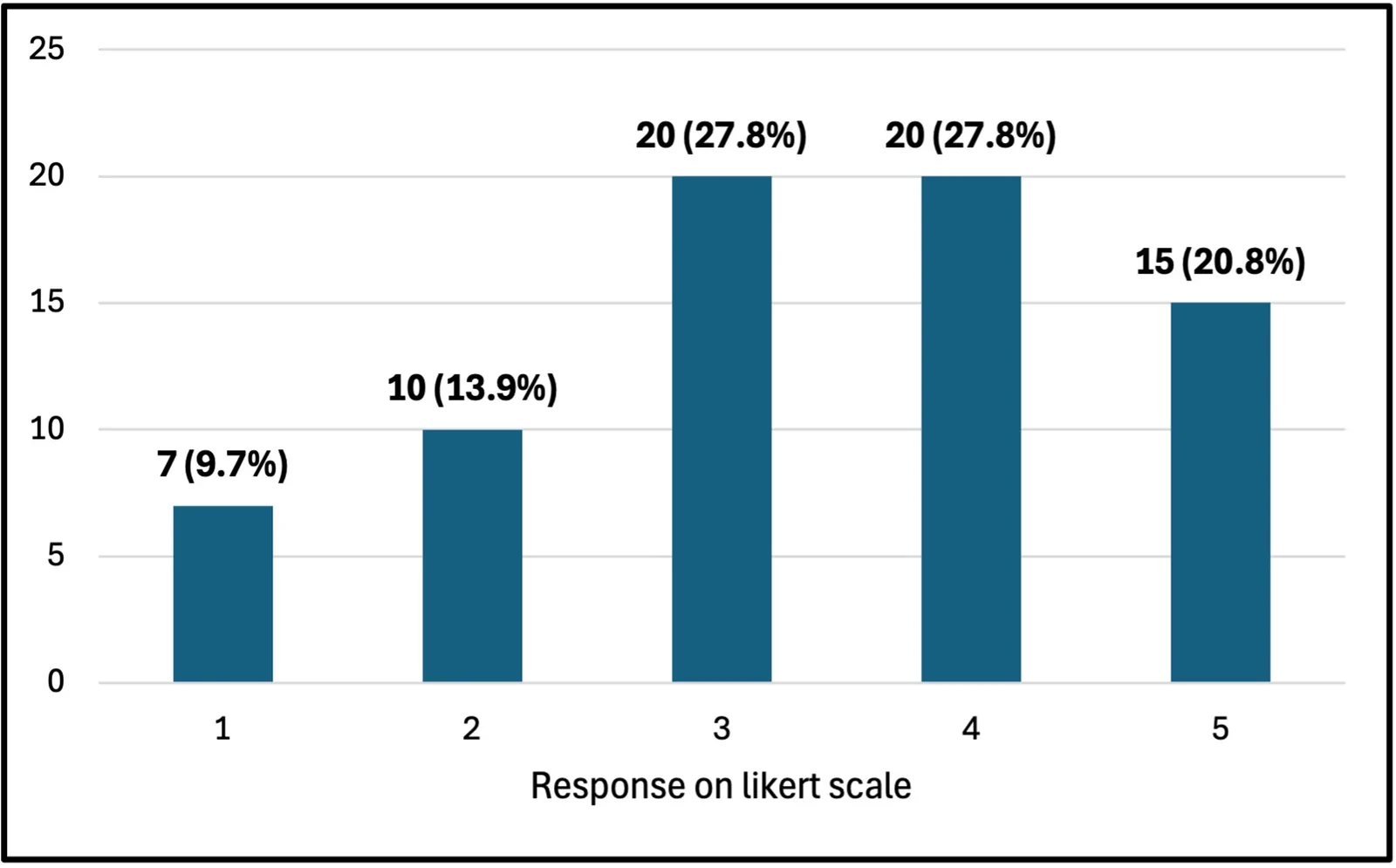
Overall distribution of Likert-scale option selection pooled across all four statements (n = 72 responses). Data presented as n (frequency).

The qualitative analysis was based on answers to the following two questions: “Describe in your own words how early exposure prepared you for Foundation Year 1?” and “Were there any specific skills, experiences, or challenges that you found particularly beneficial?”

The responses from 19 participants revealed early on-call exposure to be generally perceived as valuable. Thematic analysis of the feedback showed that participants gained insight into the responsibilities of a resident doctor during on-call shifts, as well as an understanding of how the functioning of hospitals can be different in the out-of-hours environment. One participant commented, “Understood the expectations of a junior doctor on call”.

Another theme that participants described was opportunities to practise clinical and professional skills, including clerking with greater autonomy, responding to bleeps, triaging, and communication with professional colleagues, while also being given exposure to acute presentations and situations requiring decision-making. One participant stated: “I learnt how to answer the bleep, communicate with other healthcare professionals, prioritise jobs and deal with clinical emergencies”, and another commented, “Lots of opportunity for dealing with acutely unwell patients with appropriate supervision".

A third theme was the development of confidence and readiness for independent practice, while furthering the clinical knowledge gained in medical school. This is reflected in the comment, “Allowed me to gain a greater understanding of the role of foundation doctors on on-call shifts, see more acute presentations and allowed me to clerk patients and make decisions for myself more independently”. This supports the quantitative analysis, which also demonstrated participants appreciating opportunities to practise their skills and develop their clinical understanding. A fourth theme was the effect of placements on coping with the demands of night shifts. Participants noted that the experience helped reduce anxiety about undertaking on-call duties as a Foundation Year 1 doctor, with one commenting that it “built confidence in seeing F1/2 reviewing patients in real time.” Challenges were also highlighted, particularly in adjusting to night schedules, with one participant reflecting on the benefit of “learning to adapt my sleep cycle.”

Some perceived limitations to on-call shift exposure were also discussed. One participant commented on how a limited clinical background was a hindrance to learning in the out-of-hours environment, stating, “I don’t feel it helped me very much. At that stage of training, without any independent experience, going on OOH shifts as a student, most of the useful information goes in one ear and out the other. It was helpful practising skills OOH; however, with regard to general knowledge, I don’t feel it made much of a difference”. This could possibly highlight that early on-call exposure might overwhelm students without sufficient prior clinical experience, thus limiting educational value.

Participants also highlighted that the learning opportunities during on-call shifts were highly dependent on the doctor being shadowed. One participant noted: “Night shifts weren’t particularly useful as a medical student as not much to do on wards and residents are incredibly tired to be able to participate in teaching,” while another reported the opposite: “Doctors had more time to teach and go through cases during an out-of-hours setting.” These contrasting experiences reflect that the educational benefit of on-call placements can vary significantly based on the engagement and workload of the doctor being shadowed.

It is worth noting that the one participant who did not attend on-call shifts as a student expressed that they did not think they would have benefited from early exposure and that the quality of the placement is dependent on the doctors supervising them, as opposed to the placement itself.

## Discussion

This study found that on-call exposure was mandatory for most exposed respondents and was predominantly scheduled in the fifth year. However, while such exposure is integrated into medical education, research indicates that its effectiveness in achieving long-term educational outcomes requires ongoing restructuring and evaluation. A narrative review by Priest et al. (2024) underscores the necessity for continuous adaptation of on-call simulation programs to enhance their perceived impact on student preparedness and professional development [14].

Additionally, respondents generally perceived early on-call exposure positively in relation to the General Medical Council Outcomes for Graduates [4], particularly in the domains of professional knowledge and skills. Nevertheless, a greater proportion of participants in this study perceived on-call shift exposure to be neutral or of limited benefit to their professional values and behaviours.

This is evidenced by the majority of participants reporting positive perceptions in the quantitative survey and by qualitative feedback highlighting increased insight into hospital functioning, understanding of different professional roles, and enhanced independent decision-making. These findings align with previous literature indicating that early clinical exposure, including simulated or real out-of-hours experiences, can enhance preparedness for the transition from medical student to junior doctor. For example, a 2016 study by Stroben in Germany involving simulated night shifts in the emergency department reported increased feelings of confidence and preparedness among students [15]. Similarly, national surveys have shown that shadowing and structured early exposure can improve readiness, particularly in areas such as confidence and clinical decision-making [5]. These findings are consistent with the idea that integrating practical, high-responsibility experiences into undergraduate curricula may help bridge the gap between theoretical knowledge and real-world clinical practice.

The findings regarding gains in knowledge and skills also resonate with established educational theories. Kolb’s experiential learning cycle, for example, describes how learners progress from concrete experience, such as hands-on on-call participation, to reflective observation with peers or educators, then to abstract conceptualisation, which can occur during and after the shift, and finally to active experimentation applied in later clinical practice [16]. In contrast, the more neutral perceptions relating to professional values and behaviours are consistent with the idea that these attributes are shaped primarily through longitudinal role-modelling and the hidden curriculum rather than short, intensive experiences. Educational theories support this interpretation: according to Lave and Wenger’s model of situated learning, professional identity emerges through sustained participation in communities of practice, not isolated encounters [17]. This theoretical framing is offered as an interpretive lens for the observed pattern of results, rather than as a framework the study was designed to test. Thus, the pattern observed in this study may reflect the different mechanisms through which knowledge, clinical skills, and professional values are acquired.

Despite these benefits, participants highlighted limitations in educational opportunities during on-call shifts, particularly at night. Consistent with previous reports, teaching and structured learning were described as less frequent during evenings and weekends compared to regular weekday hours [18]. Some respondents noted, however, that doctors occasionally had more time to teach during out-of-hours shifts, suggesting that the educational value of on-call experiences is highly dependent on the supervising clinician. This variability was reflected throughout the responses, with participants emphasising that the usefulness of placements often hinged on the engagement and guidance of the doctor they shadowed. Such findings underscore the importance of providing clear expectations and, where possible, assigning dedicated supervisors to maximise the educational value of on-call placements.

When integrating the quantitative survey findings with the qualitative feedback, a more nuanced picture of participants’ perceptions of on-call shifts emerges. Participants who rated the on-call shifts positively emphasised that these experiences helped them understand the National Health Service out-of-hours structure, observe different staff roles, and gain insight into the expectations placed on junior doctors. They reported that practical exposure to tasks such as clerking, triaging, responding to bleeps, and managing acute situations fostered both skill development and confidence. Those with neutral responses often indicated that the shifts primarily assisted in adjusting to night schedules and familiarising themselves with hospital operations rather than directly enhancing preparedness. These findings align with a study conducted on third-year medical students in North Carolina, which found that sleep deprivation did not impact cognitive performance, but students felt more alert and prepared for clinical duties after night shifts [19]. Conversely, participants with negative perceptions felt that on-call shifts were less useful due to limited teaching opportunities or an overwhelming volume of information, although they acknowledged that practical skills were still gained.

The mixed-methods design strengthened the study by allowing triangulation between quantitative scores and qualitative feedback, providing a more nuanced understanding of participant experiences. Conducting the survey during the transition from Foundation Year 1 to Year 2 also allowed participants to reflect on nearly a year of practical experience.

This study had several limitations. Most notably, there was no meaningful comparison group of graduates without early on-call exposure, and only one respondent reported no such exposure. As a result, the findings cannot establish a causal effect of on-call exposure on preparedness or on attainment of the GMC Outcomes for Graduates. Related to this, because on-call exposure was mandatory for most respondents (15/18) and optional for a minority (3/18), whether mandatory versus optional status moderated perceived benefit could not be meaningfully explored with this sample.

The sample was small and single-site (19 of 71 invited, 26.7% response rate), which limits the precision of the descriptive quantitative findings, constrains the transferability of the qualitative themes, and may not be generalisable to Foundation doctors trained under different undergraduate curricula or working within other Foundation Schools [20]. The response rate also raises the possibility of selection or non-response bias, as doctors with stronger views, whether positive or negative, about their undergraduate on-call experience may have been more likely to respond. Responses may additionally have been affected by the demanding and unpredictable schedules of Foundation doctors, which likely limited the time available to complete the survey despite the lottery incentive offered.

The questionnaire was investigator-developed and was not piloted or psychometrically validated, and it did not capture the number, duration, type, or specialty of on-call shifts, so the extent of exposure could not be characterised beyond academic year and mandatory/optional status. This limits the precision with which perceived benefits can be linked to specific features of exposure. The reliance on self-reported data is subject to recall bias, as participants were reflecting on experiences from up to five years earlier, and to social desirability bias, particularly for items relating to professional values and behaviours. Finally, qualitative coding was performed by a single researcher, without independent verification, which may have introduced interpretive bias. As the researcher is a doctor familiar with the Foundation training transition, this background may have further shaped interpretation of participants' responses.

Future research should investigate the optimal timing, structure, and specialties for early on-call placements, whether mandatory versus optional participation influences perceived benefit, and the potential benefits of assigning dedicated supervisors. Studies that capture the number, duration, type, and specialty of on-call shifts would also allow the characteristics of exposure to be linked more precisely to perceived outcomes. Additionally, medical schools could consider formalising on-call experiences within curricula to standardise exposure, which may support perceived preparedness.

## Conclusions

This exploratory study found that Foundation doctors generally perceived undergraduate on-call exposure as beneficial, particularly in relation to the development of clinical knowledge and professional skills, although the quantitative findings did not demonstrate statistical significance. Perceived benefits for professional values and behaviours were more limited, which may reflect the influence of longitudinal role-modelling and the hidden curriculum. The educational value of on-call exposure also appeared to be shaped by contextual factors such as shift intensity, variability in supervision, and opportunities for teaching. Educational theories suggest that while short, high-responsibility experiences may build skills, professional identity develops through sustained clinical engagement. Future research should investigate the optimal timing, structure, and supervision of undergraduate on-call experiences within medical curricula, whether mandatory versus optional participation influences perceived benefit, and how characteristics such as shift duration, type, and specialty relate to perceived educational value.

## Appendices

**Table 2:** Questionnaire used for data collection.

Question/information	Response options	Routing/logic
Description: This study explores how early exposure to on-call shifts during a UK undergraduate medical programme impacts the General Medical Council (GMC) outcomes for graduates. Specifically, it looks at the influence on professional values and behaviour, skills, and knowledge development. For more information, please read the Participant Information Sheet: https://bit.ly/4j0ZWwm. Incentive for Participation: Participants in this study will be entered into a random lottery for a chance to win one of three Amazon vouchers. The prizes are as follows: 1st Prize: £60; 2nd Prize: £40; 3rd Prize: £20. * Required	—	—
I have read and understood the information provided about this study. I understand that my participation is voluntary, that the data collected will remain anonymous (unless I choose to enter the lottery), and that I may withdraw at any time. I consent to participate in this research study and agree that my responses may be used in the research report and future publications. *	Yes, I agree to participate in this study.; No, I do not agree to participate in this study.	—
Would you like to participate in the lottery? *	Yes; No	—
If yes, please provide your email below.	Enter your answer	Displayed only if “Yes” is selected for participation in the lottery.
Kindly confirm that you are a UK medical graduate in order to participate in this survey. *	I confirm that I am a UK medical graduate.; I am not a UK medical graduate.	If “I am not a UK medical graduate” is selected, the survey ends.
During your medical studies, did you participate in any on-call shifts (including night shifts)? *	Yes; No	If “Yes” is selected, proceed to the questions concerning on-call shift experience. If “No” is selected, proceed to the questions concerning whether early exposure would have better prepared the participant for Foundation Year 1.
Was it mandatory or optional? *	Mandatory; Optional	Displayed only if “Yes” is selected for participation in on-call shifts.
In which year did this exposure occur? (Select as many as appropriate.) *	Year 1; Year 2; Year 3; Year 4; Year 5; Other	Displayed only if “Yes” is selected for participation in on-call shifts.
If you selected “Other”, please specify.	Enter your answer	Displayed only if “Other” is selected.
To what extent do you agree with the following statement about your early on-call shift experience? (Scale: 1 = Strongly Disagree to 5 = Strongly Agree) * Early on-call shift exposure during medical school increased my preparedness for Foundation Year 1.	1–5	Displayed only if “Yes” is selected for participation in on-call shifts.
To what extent do you agree with the following statement about your early on-call shift experience? (Scale: 1 = Strongly Disagree to 5 = Strongly Agree) * Early exposure to on-call shifts positively shaped my professional values and behaviour.	1–5	Displayed only if “Yes” is selected for participation in on-call shifts.
To what extent do you agree with the following statement about your early on-call shift experience? (Scale: 1 = Strongly Disagree to 5 = Strongly Agree) * Early exposure to on-call shifts contributed to the development of my professional skills.	1–5	Displayed only if “Yes” is selected for participation in on-call shifts.
To what extent do you agree with the following statement about your early on-call shift experience? (Scale: 1 = Strongly Disagree to 5 = Strongly Agree) * Early exposure to on-call shifts enhanced my professional knowledge.	1–5	Displayed only if “Yes” is selected for participation in on-call shifts.
Describe in your own words how early exposure prepared you for Foundation Year 1. *	Enter your answer	Displayed only if “Yes” is selected for participation in on-call shifts.
Were there any specific skills, experiences, or challenges that you found particularly beneficial? *	Enter your answer	Displayed only if “Yes” is selected for participation in on-call shifts.
Do you think that having early exposure to on-call shifts would have better prepared you for Foundation Year 1? *	Yes; No	Displayed only if “No” is selected for participation in on-call shifts.
Please explain why you feel early on-call exposure would or would not have helped you prepare for Foundation Year 1. *	Enter your answer	Displayed only if “No” is selected for participation in on-call shifts.
